# Prevalence of Metabolic Syndrome and Its Association with Cardiovascular Outcomes in Hospitalized Patients with Inflammatory Bowel Disease

**DOI:** 10.3390/jcm13226908

**Published:** 2024-11-16

**Authors:** Ryan Njeim, Sai Shanmukha Sreeram Pannala, Nadim Zaidan, Toni Habib, Medha Rajamanuri, Elie Moussa, Liliane Deeb, Suzanne El-Sayegh

**Affiliations:** 1 Department of Medicine, Northwell Staten Island University Hospital, Staten Island, NY 10305, USA; rnjeim@northwell.edu (R.N.); spannala@northwell.edu (S.S.S.P.); nzaidan@northwell.edu (N.Z.); thabib1@northwell.edu (T.H.); emoussa@northwell.edu (E.M.); 2 School of Medicine, Southern Illinois University, Springfield, IL 62702, USA; 3 Department of Medicine, Division of Gastroenterology and Hepatology, Northwell Staten Island University Hospital, Staten Island, NY 10305, USA; ldeeb1@northwell.edu; 4 Department of Medicine, Division of Nephrology, Northwell Staten Island University Hospital, Staten Island, NY 10305, USA

**Keywords:** inflammatory bowel disease, metabolic syndrome, hypertension, diabetes, obesity, arrhythmia, heart failure, acute coronary syndrome

## Abstract

**Background**: Patients with autoimmune diseases experience a higher burden of metabolic syndrome (MetS) and cardiovascular disease (CVD). There is a paucity of data regarding MetS in patients with inflammatory bowel disease (IBD) and its impact on CVD. In this retrospective study, we aimed to evaluate the prevalence of MetS components in IBD patients, as well as their association with acute coronary syndrome (ACS), heart failure and arrhythmias. **Methods**: After pooling 5 years of data from the National Inpatient Sample (NIS) Database (2016–2020), we compared traditional cardiovascular risk factors between IBD and non-IBD patients. We then investigated the association between MetS (represented by a calculated metabolic score (CMS) ranging from 0 to 4, based on the presence or absence of hypertension, obesity, dyslipidemia and type II diabetes) and CVD, separately for Crohn’s disease (CD) and ulcerative colitis (UC) patients. **Results**: The prevalence of the different MetS components was found to be lower in IBD patients compared to non-IBD patients. Comparing CD (n = 806,875) and UC (n = 575,925) identified a higher prevalence of MetS components in UC. Higher CMS was positively associated with ACS and arrhythmias in both CD and UC. This association was evident in heart failure, with the odds ratio increasing from 2.601 for CMS = 1 to 6.290 for CMS = 4 in UC patients and from 2.622 to 5.709 in CD patients. **Conclusions**: Our study highlights the positive association between traditional components of MetS and CVD in IBD patients. Our findings suggest that chronic inflammation explains only partially the CVD burden in hospitalized IBD patients.

## 1. Introduction

Inflammatory bowel diseases (IBD) are a group of pathologies characterized by a chronic immune-mediated inflammation of the gastrointestinal (GI) tract [1]. Genetic susceptibility [2] and environmental triggers, especially dietary factors [3], are actively being researched in this group of conditions that are increasing in prevalence [4]. IBD includes two main phenotypes: Crohn’s disease (CD) and ulcerative colitis (UC) [5]. While CD is typically defined as a transmural inflammation with skipped lesions, that can affect any part of the GI tract with notable abscess formation, fistulous tracts and strictures, inflammation in UC is continuous, limited to the mucosal layer and affects the colon only [6]. Both phenotypes share a similar clinical presentation (such as hematochezia, chronic watery diarrhea, abdominal pain and weight loss), can be associated with extra-intestinal manifestations and are considered a systemic disease [7].

Metabolic syndrome (MetS) is defined by multiple metabolic abnormalities, including hypertension, dyslipidemia, insulin resistance and central obesity, and increases the risk of cardiovascular disease (CVD) and type 2 diabetes [8,9]. The American Heart Association and Updated United States National Cholesterol Education Program Adult Treatment Panel III suggest that a diagnosis of MetS can be made with three out of five criteria, based on elevated fasting glucose or the use anti-hyperglycemic medication, elevated serum levels of triglycerides and high density lipoprotein (HDL) cholesterol, elevated blood pressure and waist circumference (>102 cm in men or >88 cm in women) [10].

MetS is strongly linked to an increased risk of cardiovascular (CV) complications as well as mortality [11]. Several studies have highlighted the role of chronic inflammation in the pathophysiology of MetS and an association was found between MetS and various autoimmune and inflammatory diseases [12,13]. With CV diseases still as the foremost cause of mortality worldwide, addressing their multifaceted contributors is essential. Several autoimmune diseases such as psoriasis, systemic lupus erythematosus, rheumatoid arthritis and vasculitis have been associated with an increased risk of CVD compared to the general population [14]. In addition to traditional risk factors, the use of immunosuppressants and steroids, on one hand, and the presence of chronic inflammation, on the other hand, have been linked to this increased risk [14,15]. In fact, it has been shown that chronic inflammation can play a pivotal role, acting as a potent catalyst for the development of accelerated atherosclerosis, promoting thrombus formation and precipitating atherosclerotic CV events [15].

The association between IBD and MetS remains controversial and poorly studied [16]. The pooled prevalence of comorbid MetS in UC has been previously found to be higher than that in Crohn’s disease (CD), a striking difference highlighting that the aggregate estimate of MetS is significantly greater in patients with UC than in those with CD [16]. While several studies have established an increased CV risk in IBD patients, the main cause of this risk remains unclear. With a lower burden of traditional CVD risk factors such as hypertension (HTN), diabetes mellitus (DM), obesity and dyslipidemia (DLD), IBD patients were found to have an increased incidence of acute coronary syndrome [17] and coronary artery disease [18]. However, the literature remains scarce when it comes to the evaluation of the impact of MetS on heart failure (HF) and arrhythmia in IBD patients.

We conducted a study using a large United States (US) database, the National Inpatient Sample (NIS) database, to investigate the burden of MetS in hospitalized IBD patients, as well as the association of single and multiple components of MetS with three CV outcomes of interest: (1) acute coronary syndrome (ACS), (2) heart failure and (3) arrhythmias. Our primary objective was to describe the prevalence of MetS components in the general hospitalized population and specifically in patients with IBD. By strictly analyzing records encoding IBD diagnoses, our secondary objective was to evaluate the association of MetS with CV outcomes while accounting for demographic and socio-economic factors in this specific cohort.

## 2. Materials and Methods

### 2.1. Data Source and Variables of Interests

The Healthcare Cost and Utilization Project’s (HCUP) NIS is maintained by the Agency for Healthcare Research and Quality (AHRQ). This US-based database compiles hospitalization records, including variables relating to patients’ characteristics, the conditions diagnosed and the procedures they underwent; variables relating to the hospitals themselves are also listed. The NIS database includes every hospitalization separately, which may result in including the same patient more than once.

The International Classification of Disease, 10th Edition (ICD-10) codes were used to identify conditions of interest, for inclusion and exclusion. The ICD10 codes used in this study are presented in Table A1 in Appendix A.

The patient’s variables of interest included age, sex, race, geographic census of the admitting hospital, income quartile and primary insurance payer. Including these covariates ensures that we are controlling for important confounders and helps us interpret the relationship between MetS and metabolic risk factors and CVD more accurately in IBD patients, especially considering that gender, racial and socioeconomic disparities exist in this population [19]. Records of patients under 18 years of age were excluded from the study.

We identified records containing an ICD10 code diagnosis for either CD or UC, and these patients constituted the IBD cohorts. Diagnoses of obesity, type II diabetes, dyslipidemia and hypertension were also identified.

For the second part of the study, ICD10 codes were also used to identify exclusion criteria. These pre-specified confounders were either genetically predetermined cardiovascular risk factors (such as type I diabetes and primary hypercholesterolemia) or autoimmune diseases such as lupus, rheumatoid arthritis, psoriasis, spondyloarthropathies, and hidradenitis suppurativa. These conditions were excluded based on their association with MetS and a higher CVD risk compared to the general population [12,13]. Additionally, studies have shown a possible link between IBD and these autoimmune diseases that can act as a confounder [20,21,22]. Patients with end-stage kidney disease were also excluded given the higher cardiovascular risk associated with advanced kidney disease compared to patients with normal or mildly reduced kidney function.

Because of the deidentified nature of the NIS database, this study is considered exempt from an Institutional Review Board (IRB) review by Northwell Health.

### 2.2. Study Design

In the first part of the study, we investigated the prevalence of MetS components in patients with and without IBD. We also compared the prevalence of CV diseases of interest, which were divided into acute coronary syndrome, heart failure and arrhythmias.

In the second half of the study, we solely focused on patients with IBD, investigating how these MetS components were associated with cardiovascular outcomes in each subpopulation, either separately or in combination. For that purpose, we first excluded patients who would specifically constitute a confounding variable, as specified above.

Then, from the pooled 5 years of NIS records (2016–2020), we extracted only those that contained a diagnosis of either UC or CD. Using the four components of metabolic syndrome cited above, which were encoded in a binary fashion (obesity, type II diabetes, dyslipidemia and hypertension), we calculated their sum in each record, yielding a calculated metabolic score (CMS), which was used as a surrogate to estimate the number of MetS parameters that each record had. This CMS ranged from 0 (indicating no components of MetS) to 4 (indicating the concomitant presence of all four components of MetS encoded in a specific record). Due to the unavailability of laboratory and clinical measurements in the NIS database (such as blood glucose, triglycerides, waist circumference, etc.), we used the CMS as a surrogate to diagnose MetS, with a score more than 2 considered positive for the diagnosis [23,24]. A regression analysis was performed accounting for this CMS, and sensitivity analyses were performed while adjusting for MetS components individually.

### 2.3. Statistical Analysis

Age is presented as means with standard error of mean, and the Wilcoxon test was used to compare both groups. Categorical variables are presented as percentages with standard errors and were compared using a second order Rao–Scott Chi-Square Test.

The complex sample feature of SAS Software 9.4 (Survey Procedures) was used for the analyses, allowing us to account for hospital stratification (NIS_STRATUM), clustering (HOSP_NIS) and discharge weights (DISCWT), which are parameters provided yearly in the NIS database.

Logistic regression was computed for strength and significance of the relationship between the CMS score and the outcome of interest. Multivariate binary logistic regression allowed us to assess whether the CMS score was independently associated with CV outcomes. A CMS of 0 was used as a reference, and regressions accounted for multiple covariates, including demographic variables such as age, sex, and race, as well as socio-economic indicators like primary expected payer, income quartile and hospital geographic division. The results are presented as odds ratio (OR) estimates with 95% confidence intervals.

A similar multivariate binary logistic regression was included in Appendix A to assess whether the presence of MetS (defined by a CMS more than 2) was independently associated with CV outcomes in both CD and UC patients. A CMS less than 2 was used as a reference, and regressions accounted for the same covariates as above.

We report statistical parameters relevant to the regression models, specifically the c-statistic and the Goodman–Kruskal Gamma. These metrics evaluate the adequacy and fitness of the models obtained by multivariate binary logistic regression.

A two-sided *p*-value of less than 0.05 was used for statistical significance.

Statistical analyses were conducted using SAS Enterprise Software, version 9.4 (SAS Institute Inc., Cary, NC, USA).

## 3. Results

### 3.1. Evaluation of Hospitalized IBD Patients Compared to the Non-IBD Patients

First, we compared hospitalized IBD patients to non-IBD patients. The IBD cohort (n = 1,488,685) exhibited distinct demographic and clinical profiles when compared to the non-IBD cohort, and the results are presented in Table 1.

The IBD patients were significantly younger with an average age of 54.51 years (*p* < 0.001). In terms of gender distribution, 56.27% of IBD patients were female, compared to 57.32% in non-IBD patients. The majority of IBD patients were White (78.9%), compared to 66.9% of non-IBD patients.

The median household income distribution was found to be different in each cohort. A smaller proportion of IBD patients were in the lowest income quartile (24.5%) compared to non-IBD patients. Regarding insurance coverage, IBD patients had a slightly lower proportion of Medicare coverage (43.4%) compared to non-IBD patients (47.0%). Additionally, IBD patients were more likely to be covered by private insurance (35.6%) compared to non-IBD patients.

The prevalence of the different components of MetS was found to be significantly lower in IBD patients. This included type II diabetes (17.50% in IBD patients compared to 25.91% in the non-IBD cohort) and dyslipidemia (23.09% of IBD patients vs. 30.70% in non-IBD patients); hypertension and obesity followed the same pattern. CV comorbidities were also notably less prevalent in IBD patients, with 9.17%, 2.44% and 1.92% of patients with arrhythmia, heart failure and acute coronary syndrome, respectively.

### 3.2. Characterization of Ulcerative Colitis and Crohn’s Disease Patients

In Table 2, after excluding potential confounding conditions, as detailed in the methods section, we extracted solely records containing an ICD10 code relative to IBD and compared CD (n = 806,875) and UC (n = 575,925) head-to-head.

In the UC cohort, the mean age was 56.35 years (95% CI: 56.17–56.52), with a gender distribution of 46.55% males and 53.44% females. The insurance was majorly covered by three primary expected payers: 43.27% by Medicare, 12.62% by Medicaid and 37.61% by private insurance. Whites represented the main ethnicity in this cohort, constituting 78%. Zip code income quartiles were 22% in the lowest quartile, 25% in the second, 26.4% in the third, and 26.5% in the highest. The CMS was as follows: 0 in 41.89%, 1 in 25.88%, 2 in 20.34%, 3 in 9.74%, and 4 in 2.12%.

Of the 806,875 hospitalized CD patients, 2470 (1.62%) had an acute myocardial infarction, 3421 (2.21%) had heart failure and 12,280 (7.92%) had arrhythmias.

Patients with UC had a higher prevalence of MetS components, leading to an overall higher CMS compared to patients with CD. Indeed, 11.88% of UC patients had a CMS above 2 compared to 9.81% in patients with CD. The prevalence of CV comorbidities was higher in UC patients, particularly for arrhythmias and acute coronary syndrome. Notably, heart failure had an almost similar prevalence, although significantly higher in UC patients (2.33% vs. 2.21% in CD patients; *p*-value = 0.04).

### 3.3. Cardiovascular Outcomes in Patients with Ulcerative Colitis

We then conducted three different multivariate binary logistic regressions to evaluate the association of MetS with cardiovascular outcomes in hospitalized patients. CMS ranged from 0 to 4, with 0 serving as the reference category; this score was a surrogate for the identification of IBD patients positive for MetS. Table 3 presents the results of these multivariate regressions, evaluating the outcomes of ACS, heart failure and arrhythmia.

The ORs for ACS in the UC cohort showed that African American patients had an OR of 0.827 (95% CI: 0.692–0.989), and females had a lower likelihood with an OR of 0.603 (95% CI: 0.553–0.657). The ORs for ACS increased with higher CMS scores compared to CMS 0: CMS 1 had an OR of 2.029 (95% CI: 1.736–2.373), rising to an OR of 3.722 (95% CI: 2.903–4.772) for CMS 4. When stratified by MetS components (Table 4), the OR for ACS was 1.707 (95% CI: 1.551–1.880) for DLD, 1.470 (95% CI: 1.308–1.652) for HTN and 1.254 (95% CI: 1.140–1.379) for type II diabetes, with only obesity not reaching significance. Additionally, Table A2 in Appendix A shows that UC patients with MetS (score > 2) had an OR of 1.739 (95% CI: 1.575–1.920) for ACS compared with those with a score < 2.

For HF, African American patients had an OR of 1.242 (95% CI: 1.073–1.437), while Hispanics had a reduced likelihood (OR: 0.764, 95% CI: 0.632–0.924), and females had an OR of 0.928 (95% CI: 0.854–1.008). The OR for HF increased with higher CMS scores compared to CMS 0: CMS 1 had an OR of 2.601 (95% CI: 2.216–3.054), CMS 2 an OR of 3.253 (95% CI: 2.759–3.836), CMS 3 an OR of 4.219 (95% CI: 3.546–5.020) and CMS 4 an OR of 6.290 (95% CI: 5.028–7.869). The OR for HF when stratified by MetS components was 2.500 (95% CI: 2.207–2.832) for HTN, 1.330 (95% CI: 1.196–1.478) for obesity, and 1.513 (95% CI: 1.385–1.651) for T2DM, with only dyslipidemia not reaching significance. The OR for HF when accounting for the presence of MetS was 1.871 (95% CI: 1.703–2.055) (Table A2 in Appendix A).

Regarding arrhythmias, African Americans had an OR of 0.796 (95% CI: 0.725–0.874) and Hispanics had an OR of 0.620 (95% CI: 0.55–0.694), while females had an OR of 0.694 (95% CI: 0.665–0.724). The ORs for arrhythmias increased with higher CMS scores from an OR of 1.541 (95% CI: 1.441–1.648) for CMS 1 to 2.566 (95% CI: 2.267–2.904) for CMS 4. The OR for arrhythmias when stratified by MetS components was 1.160 (95% CI: 1.109–1.213) for DLD, 1.553 (95% CI: 1.472–1.638) for HTN, 1.431 (95% CI: 1.348–1.520) for obesity, and 1.056 (95% CI: 1.005–1.109) for T2DM. The OR for arrhythmias when accounting for the presence of MetS was 1.457 (95% CI: 1.381–1.537) (Table A2 in Appendix A).

### 3.4. Cardiovascular Outcomes in Patients with Crohn’s Disease

This analysis was finally conducted in patients with CD, also employing three multivariate binary regressions to evaluate the association of MetS with the outcomes of ACS, heart failure and arrhythmias, as presented in Table 5.

In patients with CD, a diagnosis of ACS was more likely made in older patients (OR = 1.041 with 95% CI 1.038–1.045 for age) and less likely in African American (OR 0.742 with 95% CI 0.628–0.877) or female (OR 0.72 with 95% CI 0.669–0.787) patients. Additionally, increasing odds ratio were found with higher CMS (from 2.024 (95% CI: 1.762–2.324) in CMS 1 up to 3.501 (95% CI: 2.772–4.423) in CMS 4). When regression was conducted while accounting for individual risk factors (Table 6), the CD patients’ odds ratios for ACS were 1.719 (95% CI: 1.565–1.888) for DLD, 1.591 (95% CI: 1.424–1.778) for HTN and 1.196 (95% CI: 1.085–1.319) for T2DM. When evaluated based on the presence or absence of MetS, the OR for ACS was 1.726 (95% CI: 1.556–1.914) (Table A3 in Appendix A).

Regarding HF, age (OR 1.035, 95% CI: 1.031–1.038) and African American ethnicity (OR 1.154, 95% CI: 1.021–1.304) had a positive association with heart failure. The OR for HF in CD patients increased with higher CMS scores compared to CMS 0: CMS 1 had an OR of 2.622 (95% CI: 2.308–2.978), rising to an OR of 5.709 (95% CI: 4.674–6.973) for CMS 4. Additionally, hypertension (OR 2.452, 95% CI 2.214–2.716), type II diabetes (OR 1.627 95% CI 1.500–1.765) and obesity (OR 1.469, 95% CI 1.338–1.612) but not dyslipidemia had a positive association with HF. The OR for HF when accounting for the presence of MetS was 2.099 (95% CI: 1.926–2.287) (Table A3 in Appendix A).

Finally, concerning arrhythmias in CD, White patients were more likely to have arrhythmias among their comorbidities (African Americans, Hispanics and Asians’ respective ORs were 0.82, 0.66, and 0.72) as were older patients (OR 1.054, 95% CI 1.052–1.056). Similarly, a higher CMS score was associated with a higher odds ratio in these patients. Moreover, CD patients with arrhythmias were more likely to have all four traditional risk factors. The OR for arrhythmias when accounting for the presence of MetS was 1.586 (95% CI: 1.505–1.671) (Table A3 in Appendix A).

## 4. Discussion

Our analysis demonstrates significant associations between MetS or its individual components and cardiovascular comorbidities in IBD patients, with notable variations across different genders and racial groups. In addition to evaluating the contribution of traditional CV risk factors, emphasis was placed on social determinants of health by including income quartiles and expected payer in our regression models. Regression fitness parameters underscore the robustness of our models and they increase confidence in the witnessed associations.

Conflicting data are present in the literature regarding CVD outcomes in IBD patients compared to non-IBD patients. While some studies suggest that IBD is associated with a higher CVD risk compared to the general population [25,26], other studies did not show any statistically significant difference in acute coronary events with IBD or only showed positive correlation between ACS and IBD in elderly patients and patients with extra-intestinal manifestation [27,28]. Additionally, IBD patients with ACS had a more favorable ACS-related risk profile and mortality when compared to non-IBD patients with ACS [17]. While our analysis of the admitted patients showed a lower incidence of cardiovascular outcomes in patients with IBD compared to non-IBD patients, it is important to note that the NIS database includes all patients with a medical history of IBD and CVD and not only patients admitted for conditions related directly to these diseases. Thus, the patients included in our analysis have IBD that can range from mild to severe disease and might not have been in flare or active inflammation at the time of admission.

Furthermore, multiple studies found that IBD patients have a lower burden of traditional CV risk factors [18,19,20,21,22,23,24,25,26,27,28,29,30]. This raises the question whether chronic inflammation potentially drives the development of atherosclerosis and eventually CVD in this population [31]. Chronic inflammation in autoimmune diseases is known to contribute to endothelial dysfunction, increased arterial stiffness and plaque formation, potentially exacerbating CVD risk. In IBD, elevated pro-inflammatory cytokines (e.g., CRP, IL-6, TNF-α) and oxidative stress contributes to endothelial cell apoptosis, vascular dysfunction and a pro-thrombotic state [32]. Similarly, in MetS, low-grade inflammation heightens atherosclerosis and insulin resistance, key factors in cardiovascular disease. Overexpression of the immune receptor CD44 activates inflammatory cells, while increased pro-inflammatory cytokines and oxidative stress exacerbate vascular endothelial activation, promoting atherosclerosis [33,34,35]. A recent meta-analysis found that IBD patients had twice the risk of having metabolic-dysfunction-associated fatty liver disease (MAFLD) compared to the general population, with up to a third of IBD patients experiencing this metabolic complication. Other studies found similar findings with increased morbidity and mortality in hospitalized IBD patients with metabolic-dysfunction–associated steatotic liver disease (MASLD) [36,37].

Ultimately, whether controlling disease activity and aiming at reducing inflammation will decrease the CVD risk remains unanswered at this point. This also raises questions about the role of aggressive management of traditional risk factors (HTN, DM, DLD and obesity) in reducing CV risk in patients with IBD.

Our results suggest that MetS plays a major role in multiple cardiovascular comorbidities, with higher CMS in UC and CD patients associated with higher odds of ACS, arrhythmias and heart failure. Moreover, when stratified based on individual risk factors, it appears that HTN had the highest OR for HF and arrhythmias in both UC (2.5 and 1.55, respectively) and CD patients (2.45 and 1.59 respectively). Whereas dyslipidemia had the highest OR (around 1.71) for ACS in both UC and CD patients.

Our study findings align with a large cohort study that demonstrated a significantly higher cumulative risk of hypertension particularly in those with UC, where the incidence was 10.9% compared to 7.7% in CD [38]. It was also shown that UC was independently associated with the subsequent development of hypertension (HR: 1.30; 95% CI: 1.11–1.52; *p* = 0.001), while no such association was observed for Crohn’s disease or unclassified IBD [38]. We also reported a prevalence of 17.50% for type II diabetes in hospitalized IBD patients, with population-based prospective cohorts of IBD patients revealing a 50% higher risk of developing diabetes compared to the general population, a risk consistently observed in both CD and UC patients, in cohorts both from Denmark and Korea [39,40]. In addition to the increased odds of hypertension and type II diabetes, IBD was associated with higher odds of dyslipidemia [41], with persistent dyslipidemia linked to an increased risk of more severe disease activity and poorer long-term outcomes in UC [42]. This mirrors the impact of diabetes on the IBD disease course with a higher risk of IBD-related hospitalization and acute flares [43]. Obesity was linked to a higher likelihood of acute flares while on biologics [44]. However, this should be held against the recent evidence indicating that UC patients with coexisting MetS tend to have clinically and endoscopically milder disease [45]. Contrary to the expectation that systemic inflammation in MetS would worsen UC, studies have shown reduced endoscopic inflammation and less severe symptoms in these patients.

In a case-control analysis using data from the INTERHEART study, researchers demonstrated that the presence of MetS, as defined by either the World Health Organization (WHO) or the International Diabetes Federation (IDF), is associated with more than a 2.5-fold increase in the risk of acute myocardial infarction (MI) [46]. Our analysis demonstrated an exponential increase in MI risk with the presence of two or more MetS components in both CD and UC patients. Of note, in both disease forms, we observed that African American patients had lower odds of experiencing MI when compared to White patients. This could be attributed to evidence showing that the severity and duration of UC tend to be milder in African American patients compared to White patients [47], with lower odds of requiring treatment with steroids, biologics or immunosuppressants such as 6-mercaptopurine and azathioprine (AZA) [48]. More research is needed to clarify this relationship, as the reduced odds of MI in African American patients could either be due to genuinely less severe UC or disparities in access to care and/or quality of care.

Multiple studies have highlighted the role of MetS as a significant risk factor for HF across different populations. In a large cohort of middle-aged men, the presence of MetS was shown to be a significant predictor of HF, even after adjusting for other risk factors such as left ventricular hypertrophy, hypertension, diabetes, body mass index (BMI), and smoking [49]. MetS independently predicted both diastolic and systolic heart failure in a study involving 347 participants, with hypertension also emerging as a significant independent factor for both forms of heart failure [50]. There are limited data exploring the combined impact of MetS and UC on HF. In our study, we observed a significant increase in the OR for HF in both CD and UC patients with a CMS of 2 or higher. One other study showed the adjusted hazard ratio for heart failure was significantly elevated in UC but not CD patients [30].

It has been reported that MetS is associated with supra-ventricular tachycardia in patients without structural heart diseases; as well, a BMI above 25 was found to be most strongly associated with atrial fibrillation and flutter among the different MetS components [51]. Similarly, in our study, obesity in IBD patients had a clear positive association with comorbid arrhythmias. Retrospective studies have shown that QTc quartiles reflect the risk of atrial fibrillation in patients with MetS [52]. MetS also emerged as a significant independent predictor of atrial arrhythmias in patients with pacemaker implantation [53]. Most commonly, arrhythmias are thought to arise from impaired autonomic cardiovascular regulation, and in conditions such as IBD, chronic inflammation may contribute to disturbances in this autonomic control, leading to rhythm abnormalities. Specifically, in UC, studies have pointed to impaired cardiac vagal control, even during periods of disease quiescence, suggesting that the inflammation inherent to UC disrupts the autonomic nervous system. Interestingly, this impaired vagal control has not been observed in patients with CD [54]. This finding could be attributed to medication use, as there have been multiple case reports suggesting that AZA used in the treatment of UC can precipitate atrial fibrillation [55]. However, the paucity of data exploring the occurrence of arrhythmias in IBD patients highlights the role of our study in triggering more research on arrhythmias and its burden in CD and UC patients.

This study has multiple limitations. The retrospective nature of the NIS database limits the inferences that can be made to associations. The acquisition of the variables of interest, based on ICD10 codes, is dependent on the accuracy of providers and could be prone to documentation errors. In the absence of laboratory values and anthropometric data, the calculated metabolic score (CMS) can only be used as a surrogate for the presence or absence of MetS in hospitalized IBD patients, and its use may not fully capture the complexity of metabolic syndrome as seen in clinic practice. Our study could not account for endoscopic IBD burden or disease activity, as well as the treatment used, whether it was traditional immunosuppressants, steroids or the new generation of biologics that could act as a confounder to the relationship between MetS and CVD. Furthermore, ICD-10 codes lack sufficient accuracy to distinguish between IBD severity levels. The NIS database includes every hospitalization separately, which may result in including the same patient more than once. However, given the large number of patients included in this database, we estimate that this limitation had minimal impact on our analysis. Additionally, our study did not specifically investigate the impact of the rural and urban distribution of patients in the statistical analysis, although this was accounted for in the stratification process. Finally, our database only included IBD patients who were hospitalized; therefore, this might not be representative of the true MetS and CVD burdens in all IBD patients, specifically those not hospitalized, who would usually have milder disease.

## 5. Conclusions

The observed increase in the odds of ACS and HF with increasing calculated metabolic scores (CMS) reinforces the cumulative impact of multiple MetS components on cardiovascular outcomes in hospitalized patients with IBD. Our study thus highlights the positive association between traditional components of MetS and CV disease in this population. The data suggest that these comorbidities play an important role in CVD development in IBD patients and that appropriate screening, risk stratification and risk control strategies are still vital in addressing CVD occurrence in this population. Our research identifies a critical need for prospective, multicenter cohorts, exploring the intricate relationship between MetS and IBD. This will be necessary to clarify the mechanisms driving these associations, to better understand how different populations are affected and to develop targeted interventions that can reduce the cardiovascular burden in this high-risk group. Encouraging future research in this area will help refine treatment strategies and improve outcomes for IBD patients with coexisting metabolic syndrome.

## Figures and Tables

**Table 1:** Demographic and Clinical Characteristics of IBD vs. Non-IBD Patients.

Characteristic		IBD Patients	Non-IBD Patients	*p*-Value
Weighted n		1,488,685	147,266,352	
Age (Mean, SE of M)		54.51 (0.06)	58.03 (0.04)	<0.0001
Sex (%)				<0.0001
	Male	43.72 (0.10)	42.67 (0.06)	
	Female	56.27 (0.10)	57.32 (0.06)	
Race (%)				<0.0001
	White	78.91 (0.18)	66.87 (0.21)	
	African American	11.03 (0.12)	15.35 (0.14)	
	Hispanic	6.17 (0.10)	11.31 (0.14)	
	Asian or Pacific Island	1.20 (0.03)	2.78 (0.05)	
	Native American	0.38 (0.01)	0.66 (0.02)	
	Other	2.28 (0.07)	3.00 (0.06)	
Primary Expected Payer (%)				<0.0001
	Medicare	43.44 (0.15)	47.47 (0.10)	
	Medicaid	14.55 (0.10)	18.52 (0.10)	
	Private Insurance	35.53 (0.17)	26.65 (0.10)	
	Self Pay	3.48 (0.05)	4.07 (0.04)	
	No charge	0.34 (0.01)	0.35 (0.01)	
	Other	2.63 (0.05)	2.91 (0.03)	
Quartile Income (%)				<0.0001
	Q1	24.53 (0.21)	30.51 (0.20)	
	Q2	25.75 (0.18)	26.37 (0.14)	
	Q3	25.74 (0.17)	23.61 (0.13)	
	Q4	23.96 (0.27)	19.49 (0.21)	
Hypertension (%)		43.73 (0.14)	52.23 (0.08)	<0.0001
Diabetes (%)		17.50 (0.09)	25.91 (0.05)	<0.0001
Obesity (%)		12.67 (0.09)	16.61 (0.06)	<0.0001
Dyslipidemia (%)		23.09 (0.11)	30.70 (0.08)	<0.0001
Arrhythmia (%)		9.17 (0.06)	11.57 (0.03)	<0.0001
Heart Failure (%)		2.44 (0.03)	3.65 (0.02)	<0.0001
Acute Coronary Syndrome (%)		1.92 (0.03)	3.40 (0.02)	<0.0001

NIS databases were first pooled from 2016 to 2020. Hospital records were categorized based on the presence or absence of inflammatory bowel disease (IBD). We first present age as mean with standard error of mean, and *p*-value is from Wilcoxon test. The rest of the covariates are presented as percentages with standard error of percentage, and they include gender, race, primary expected payer, and income quartile, as well as traditional cardiovascular risk factors that are components of the metabolic syndrome, such as type II diabetes, dyslipidemia, hypertension and obesity. Prevalence of cardiovascular comorbidities are included in the table. *p*-values are from second order chi-square (Rao–Scott Chi-Square Test).

**Table 2:** Comparison of patients with Crohn’s disease and ulcerative colitis.

Characteristic		Crohn’s Disease	Ulcerative Colitis	*p*-Value
Weighted n		806,875	575,925	
Age (Mean, SE of M)		52.56 (0.07)	56. 35 (0.09)	
Sex (%)				<0.0001
	Male	42.48 (0.14)	46.56 (0.16)	
	Female	57.52 (0.14)	53.44 (0.16)	
Race (%)				<0.0001
	White	80.06 (0.19)	78.02 (0.22)	
	African American	11.68 (0.14)	9.29 (0.13)	
	Hispanic	4.97 (0.10)	7.90 (0.14)	
	Asian or Pacific Island	0.88 (0.03)	1.68 (0.05)	
	Native American	0.35 (0.2)	0.40 (0.02)	
	Other	2.04 (0.07)	2.70 (0.10)	
Primary Expected Payer (%)				<0.0001
	Medicare	41.26 (0.16)	43.27 (0.20)	
	Medicaid	16.40 (0.13)	12.62 (0.13)	
	Private Insurance	35.51 (0.19)	37.61 (0.22)	
	Self Pay	3.76 (0.06)	3.43 (0.07)	
	No charge	0.38 (0.02)	0.33 (0.02)	
	Other	2.69 (0.06)	2.72 (0.06)	
Quartile Income (%)				<0.0001
	Q1	26.08 (0.23)	22.03 (0.22)	
	Q2	26.25 (0.19)	25.04 (0.21)	
	Q3	25.40 (0.18)	26.41 (0.20)	
	Q4	22.26 (0.27)	26.52 (0.31)	
Hypertension (%)		42.32 (0.16)	46.68 (0.20)	<0.0001
Diabetes (%)		15.64 (0.10)	18.42 (0.13)	<0.0001
Obesity (%)		12.28 (0.10)	12.66 (0.12)	0.0041
Dyslipidemia (%)		19.79 (0.12)	26.57 (0.17)	<0.0001
Calculated Metabolic Score (%)				<0.0001
	0	47.27 (0.17)	41.89 (0.20)	
	1	26.63 (0.12)	25.88 (0.14)	
	2	16.68 (0.10)	20.34 (0.13)	
	3	7.60 (0.07)	9.75 (0.10)	
	4	1.81 (0.03)	2.13 (0.04)	
Arrhythmia (%)		7.92 (0.08)	10.25 (0.10)	<0.0001
Heart Failure (%)		2.21 (0.04)	2.33 (0.05)	0.0409
Acute Coronary Syndrome (%)		1.62 (0.03)	2.13 (0.04)	<0.0001

After pooling NIS databases between 2016 and 2020, records encoding ICD10 codes relative to IBD were extracted and records containing any of the confounding covariates specified in the Methods section were excluded. We present age as mean with standard error of percentage; meanwhile, all other covariates are presented as percentages with standard error of percentage. These include gender, race, primary expected payer, and income quartile, as well as traditional cardiovascular risk factors that are components of metabolic syndrome, such as type II diabetes, dyslipidemia, hypertension and obesity. A calculated metabolic score (CMS) was computed based on the number of risk factors identified in each record. Prevalence of cardiovascular comorbidities are included in the table. *p*-values are from second order chi-square (Rao–Scott Chi-Square Test).

**Table 3:** Association of metabolic syndrome with cardiovascular outcomes of interests in patients with ulcerative colitis.

		Odds Ratio Point Estimate [95% CI]		
Covariates		ACS	HF	Arrhythmia
Age		1.040 [1.036–1.044]	1.038 [1.033–1.043]	1.052 [1.049–1.054]
Race (Ref: White)	African American	0.827 [0.692–0.989]	1.242 [1.073–1.437]	0.796 [0.725–0.874]
	Hispanics	0.900 [0.742–1.091]	0.764 [0.632–0.924]	0.620 [0.554–0.694]
	Asian or Pacific Islander	0.875 [0.612–1.251]	0.676 [0.448–1.022]	0.698 [0.578–0.843]
	Native American	1.026 [0.478–2.202]	0.896 [0.418–1.921]	0.966 [0.652–1.431]
	Other Ethnicity	0.807 [0.587–1.111]	1.043 [0.785–1.385]	0.797 [0.673–0.944]
Female (Ref: Male)		0.603 [0.553–0.657]	0.928 [0.854–1.008]	0.694 [0.665–0.724]
Hospital Census Division		1.007 [0.989–1.025]	1.038 [1.021–1.056]	0.996 [0.987–1.005]
Primary Expected Payer (Ref: Medicare)	Pay: Medicaid	1.203 [0.979–1.478]	1.190 [0.975–1.452]	0.835 [0.747–0.935]
	Pay: Private insurance	1.164 [1.026–1.321]	0.715 [0.615–0.831]	0.836 [0.781–0.896]
	Pay: Self-pay	1.159 [0.817–1.644]	0.846 [0.583–1.228]	0.568 [0.456–0.709]
	Pay: No charge	1.232 [0.459–3.308]	1.466 [0.655–3.284]	0.823 [0.469–1.447]
	Pay: Other	1.059 [0.786–1.427]	0.768 [0.545–1.083]	0.842 [0.718–0.988]
ZIPINC (Ref: 1st–25th perc.)	ZIPINC: 26–50th perc.	0.990 [0.877–1.117]	0.815 [0.727–0.913]	1.056 [0.990–1.127]
	ZIPINC: 51st–75th perc.	0.814 [0.720–0.921]	0.672 [0.596–0.756]	1.076 [1.009–1.147]
	ZIPINC: 76–100th perc.	0.861 [0.760–0.974]	0.655 [0.580–0.739]	1.066 [0.999–1.138]
CMS score (Ref: 0)	CMS score 1	2.029 [1.736–2.373]	2.601 [2.216–3.054]	1.541 [1.441–1.648]
	CMS score 2	2.638 [2.249–3.094]	3.253 [2.759–3.836]	1.909 [1.783–2.045]
	CMS score 3	3.404 [2.871–4.037]	4.219 [3.546–5.020]	2.172 [2.011–2.346]
	CMS score 4	3.722 [2.903–4.772]	6.290 [5.028–7.869]	2.566 [2.267–2.904]
c-statistic		0.746	0.767	0.783
Goodman–Kruskal Gamma		0.504	0.544	0.569

We present the results of multivariate binary logistic regressions evaluating the association of calculated metabolic syndrome score with three outcomes of interests (ACS, HF and arrhythmia) in hospitalized patients with ulcerative colitis. Regressions are adjusted to age, ethnicity, gender, primary expected payer, hospital geographic census and median household income. Results are presented as odds ratios with 95% confidence intervals. CMS score: calculated metabolic syndrome score, ACS: acute coronary syndrome, HF: heart failure, ZIPINC: median household income for patient’s ZIP code, perc.: percentile.

**Table 4:** Traditional risk factor association with cardiovascular outcomes in hospitalized ulcerative colitis patients.

	Odds Ratio Estimate [95% Confidence Interval]		
	ACS	HF	Arrhythmia
Dyslipidemia	1.707 [1.551–1.880]	1.037 [0.951–1.131]	1.160 [1.109–1.213]
Hypertension	1.470 [1.308–1.652]	2.500 [2.207–2.832]	1.553 [1.472–1.638]
Obesity	1.070 [0.949–1.208]	1.330 [1.196–1.478]	1.431 [1.348–1.520]
Diabetes	1.254 [1.140–1.379]	1.513 [1.385–1.651]	1.056 [1.005–1.109]
c-statistic	0.750	0.772	0.784
Goodman–Kruskal Gamma	0.512	0.554	0.571

Odds ratios for traditional risk factors in hospitalized ulcerative colitis patients with regard to ACS, HF and arrhythmia. These ORs with 95% confidence interval resulted from multivariate binary logistic regression accounting from age, ethnicity, gender, primary expected payer, median household income and hospital geographic censes. ACS: acute coronary syndrome, HF: heart failure.

**Table 5:** Association of metabolic syndrome with cardiovascular outcomes of interest in patients with Crohn’s disease.

		Odds Ratio Point Estimate [95% CI]		
Covariates		ACS	HF	Arrhythmia
Age		1.041 [1.038–1.045]	1.035 [1.031–1.038]	1.054 [1.052–1.056]
Race (Ref: White)	African American	0.742 [0.628–0.877]	1.154 [1.021–1.304]	0.788 [0.724–0.858]
	Hispanics	0.853 [0.683–1.066]	0.845 [0.694–1.029]	0.654 [0.577–0.741]
	Asian or Pacific Islander	1.067 [0.665–1.713]	0.615 [0.366–1.035]	0.717 [0.559–0.921]
	Native American	1.488 [0.799–2.768]	1.455 [0.901–2.350]	0.812 [0.529–1.247]
	Other Ethnicity	1.069 [0.787–1.453]	0.997 [0.751–1.323]	0.920 [0.782–1.081]
Female (Ref: Male)		0.725 [0.669–0.787]	0.972 [0.903–1.046]	0.762 [0.731–0.794]
Hospital Census Division		0.989 [0.970–1.007]	1.043 [1.027–1.059]	1.003 [0.993–1.012]
Primary Expected Payer (Ref: Medicare)	Pay: Medicaid	1.235 [1.041–1.465]	1.192 [1.028–1.383]	0.905 [0.823–0.995]
	Pay: Private insurance	1.047 [0.927–1.183]	0.553 [0.487–0.628]	0.859 [0.809–0.912]
	Pay: Self-pay	1.651 [1.267–2.151]	0.700 [0.514–0.995]	0.798 [0.670–0.950]
	Pay: No charge	0.768 [0.244–2.417]	1.274 [0.651–2.494]	0.695 [0.401–1.203]
	Pay: Other	1.355 [1.047–1.752]	0.987 [0.777–1.253]	0.875 [0.751–1.018]
ZIPINC (Ref: 1st–25th perc.)	ZIPINC: 26–50th perc.	0.869 [0.777–0.973]	0.855 [0.778–0.940]	1.029 [0.970–1.091]
	ZIPINC: 51st–75th perc.	0.853 [0.762–0.956]	0.705 [0.637–0.780]	1.066 [1.005–1.130]
	ZIPINC: 76–100th perc.	0.711 [0.628–0.805]	0.603 [0.538–0.675]	1.089 [1.024–1.158]
CMS score (Ref: 0)	CMS score 1	2.024 [1.762–2.324]	2.622 [2.308–2.978]	1.674 [1.578–1.775]
	CMS score 2	2.866 [2.486–3.304]	3.716 [3.248–4.252]	2.152 [2.023–2.289]
	CMS score 3	3.365 [2.872–3.943]	4.998 [4.331–5.767]	2.425 [2.257–2.605]
	CMS score 4	3.501 [2.772–4.423]	5.709 [4.674–6.973]	3.121 [2.786–3.495]
c-statistic		0.759	0.782	0.795
Goodman–Kruskal Gamma		0.534	0.575	0.593

We present the results of three multivariate binary logistic regressions evaluating the association of calculated metabolic syndrome score in hospitalized Crohn’s disease patients with three outcomes of interest: ACS, HF and arrhythmia. Regressions are adjusted to age, ethnicity, gender, primary expected payer, hospital geographic census and median household income. Results are presented as odds ratios with 95% confidence intervals. CMS score: calculated metabolic syndrome score, ACS: acute coronary syndrome, HF: heart failure, ZIPINC: median household income for patient’s ZIP code, perc.: percentile.

**Table 6:** Traditional risk factor association with cardiovascular outcomes in hospitalized Crohn’s disease patients.

	Odds Ratio Estimate [95% Confidence Interval]		
	ACS	HF	Arrhythmia
Dyslipidemia	1.719 [1.565–1.888]	1.039 [0.959–1.125]	1.312 [1.255–1.372]
Hypertension	1.591 [1.424–1.778]	2.452 [2.214–2.716]	1.589 [1.513–1.669]
Obesity	1.091 [0.968–1.230]	1.469 [1.338–1.612]	1.427 [1.348–1.511]
Diabetes	1.196 [1.085–1.319]	1.627 [1.500–1.765]	1.057 [1.007–1.109]
c-statistic	0.763	0.786	0.795
Goodman–Kruskal Gamma	0.542	0.583	0.593

Odds ratios for traditional risk factors in hospitalized Crohn’s disease patients with regard to ACS, HF and arrhythmia. These ORs with 95% confidence interval are the result of multivariate binary logistic regression accounting to age, ethnicity, gender, primary expected payer, median household income and hospital geographic censes. ACS: acute coronary syndrome, HF: heart failure.

## Data Availability

Request for accessing the NIS database can be placed with AHRQ.

## Appendix A

**Table A1:** ICD 10 scores used for data extraction from the NIS database.

Variable of Interest	ICD10 Codes Used
Type II Diabetes	‘E1100’, ‘E1101’, ‘E1110’, ‘E1111’, ‘E1121’, ‘E1122’, ‘E1129’, ‘E11311’, ‘E11319’, ‘E11321’, ‘E113211’, ‘E113212’, ‘E113213’, ‘E1132199’, ‘E11329’, ‘E113291’, ‘E113292’, ‘E113293’, ‘E113299’, ‘E11331’, ‘E113311’, ‘E113312’, ‘E113313’, ‘E113319’, ‘E11339’, ‘E113391’, ‘E113392’, ‘E113393’, ‘E113399’, ‘E11341’, ‘E113411’, ‘E113412’, ‘E113413’, ‘E113419’, ‘E11349’, ‘E113491’, ‘E113492’, ‘E113493’, ‘E113499’, ‘E11351’, ‘E113511’, ‘E113512’, ‘E113513’, ‘E113519’, ‘E113521’, ‘E113522’, ‘E113523’, ‘E113529’, ‘E113531’, ‘E113532’, ‘E113533’, ‘E113539’, ‘E113541’, ‘E113542’, ‘E113543’, ‘E113549’, ‘E113551’, ‘E113552’, ‘E113553’, ‘E113559’, ‘E11359’, ‘E113591’, ‘E113592’, ‘E113593’, ‘E113599’, ‘E1136’, ‘E1137X1’, ‘E1137X2’, ‘E1137X3’, ‘E137X9’, ‘E1139’, ‘E1140’, ‘E1141’, ‘E1142’, ‘E1143’, ‘E1144’, ‘E1149’, ‘E1151’, ‘E1152’, ‘E1159’, ‘E11610’, ‘E11618’, ‘E11620’, ‘E11621’, ‘E11622’, ‘E11628’, ‘E11630’, ‘E11638’, ‘E11641’, ‘E11649’, ‘E1165’, ‘E1169’, ‘E118’, ‘E119’)
Dyslipidemia	‘E785’, ‘E780’, ‘E781’, ‘E782’, ‘E783’, ‘E784’
Obesity	‘E6601’, ‘E6609’, ‘E662’, ‘E663’, ‘E668’, ‘E669’
Hypertension	‘I10’, ‘I110’, ‘I119’, ‘I129’, ‘I130’
Crohn’s Disease	K5000’, ‘K50011’, ‘K50012’, ‘K50013’, ‘K50014’, ‘K50018’, ‘K50019’, ‘K50100’, ‘K50111’, ‘K50112’, ‘K50113’, ‘K50114’, ‘K50118’, ‘K50119’, ‘K5080’, ‘K508011’, ‘K508012’, ‘K508013’, ‘K508014’, ‘K508018’, ‘K508019’, ‘K5090’, ‘K50911’, ‘K50912’, ‘K50913’, ‘K50914’, ‘K50918’, ‘K50919’
Ulcerative Colitis	‘K5100’, ‘K51011’, ‘K51012’, ‘K51013’, ‘K51014’, ‘K51018’, ‘K51019’, ‘K5120’, ‘K51211’, ‘K51212’, ‘K51213’, ‘K51214’, ‘K51218’, ‘K51219’, ‘K5130’, ‘K51311’, ‘K51312’, ‘K51313’, ‘K51314’, ‘K51318’, ‘K51319’, ‘K5130’, ‘K51311’, ‘K51312’, ‘K51313’, ‘K51314’, ‘K51318’, ‘K51319’, ‘K5140’, ‘K51411’, ‘K51412’, ‘K51413’, ‘K51414’, ‘K51418’, ‘K51419’, ‘K5150’, ‘K51511’, ‘K51512’, ‘K51513’, ‘K51514’, ‘K51518’, ‘K51519’, ‘K5180’, ‘K51811’, ‘K51812’, ‘K51813’, ‘K51814’, ‘K51818’, ‘K51819’, ‘K5190’, ‘K51911’, ‘K51912’, ‘K51913’, ‘K51914’, ‘K51918’, ‘K51919’
Heart Failure	‘I500’, ‘I501’, ‘I502’, ‘I503’, ‘I504’, ‘I505’, ‘I506’, ‘I507’, ‘I508’, ‘I509’
Acute Coronary Syndrome	‘I2101’, ‘I2102’, ‘I2109’, ‘I214’, ‘I219’, ‘I21A1’, ‘I21A9’, ‘I220’, ‘I221’, ‘I222’, ‘I228’, ‘I229’
Arrhythmia	‘I470’, ‘I471’, ‘I472’, ‘I479’, ‘I480’, ‘I481’, ‘I482’, ‘I483’, ‘I484’, ‘I489’, ‘I490’, ‘I491’, ‘I492’, ‘I493’, ‘I494’, ‘I495’, ‘I498’, ‘I499’
ESKD	‘N185’, ‘N186’
Type I Diabetes	‘E101’, ‘E102’, ‘E103’, ‘E104’, ‘E105’, ‘E106’, ‘E107’, ‘E108’, ‘E109’
Lupus	‘L930’, ‘L931’, ‘L932’, ‘L940’, ‘L941’, ‘L942’, ‘L943’, ‘L944’, ‘L945’, ‘L946’, ‘L947’, ‘L948’, ‘L949’
Rheumatoid Arthritis	‘M0500’, ‘M051’, ‘M052’, ‘M053’, ‘M054’, ‘M055’, ‘M056’, ‘M057’, ‘M058’, ‘M059’, ‘M0600’, ‘M061’, ‘M062’, ‘M063’, ‘M064’, ‘M065’, ‘M066’, ‘M067’, ‘M068’, ‘M069’
Psoriasis	‘L400’, ‘L401’, ‘L402’, ‘L403’, ‘L404’, ‘L405’, ‘L406’, ‘L407’, ‘L408’, ‘L409’
Spondylarthropathies	‘M450’, ‘M451’, ‘M452’, ‘M453’, ‘M454’, ‘M455’, ‘M456’, ‘M457’, ‘M458’, ‘M459’, ‘M4681’, ‘M4681’, ‘M4682’, ‘M4683’, ‘M4684’, ‘M4685’, ‘M4686’, ‘M4687’, ‘M4688’, ‘M4689’, ‘M4690’, ‘M4691’, ‘M4692’, ‘M4693’, ‘M4694’, ‘M4695’, ‘M4696’, ‘M4697’, ‘M4698’, ‘M4699’
Hidradenitis suppurativa	‘L732’
Primary Hypercholesterolemia	‘E7801’

**Table A2:** Association between metabolic syndrome (CMS > 2) and cardiovascular outcomes in ulcerative colitis patients.

		Odds Ratio Point Estimate [95% CI]		
Covariates		ACS	HF	Arrhythmia
Age		1.048 [1.045–1.052]	1.047 [1.043–1.051]	1.057 [1.055–1.059]
Race (Ref: White)	African American	0.864 [0.723–1.033]	1.307 [1.128–1.514]	0.823 [0.749–0.904]
	Hispanics	0.898 [0.740–1.089]	0.760 [0.629–0.920]	0.622 [0.555–0.696]
	Asian or Pacific Islander	0.870 [0.609–1.244]	0.666 [0.440–1.006]	0.696 [0.577–0.841]
	Native American	1.017 [0.476–2.175]	0.888 [0.414–1.904]	0.959 [0.647–1.422]
	Other Ethnicity	0.794 [0.577–1.093]	1.018 [0.768–1.351]	0.789 [0.667–0.933]
Female (Ref: Male)		0.591 [0.542–0.644]	0.912 [0.839–0.990]	0.684 [0.655–0.713]
Hospital Census Division		1.005 [0.987–1.023]	1.035 [1.018–1.053]	0.994 [0.986–1.003]
Primary Expected Payer (Ref: Medicare)	Pay: Medicaid	1.184 [0.961–1.458]	1.152 [0.940–1.411]	0.818 [0.731–0.915]
	Pay: Private insurance	1.135 [0.997–1.292]	0.683 [0.585–0.798]	0.814 [0.759–0.872]
	Pay: Self-pay	1.078 [0.759–1.530]	0.762 [0.524–1.108]	0.537 [0.430–0.669]
	Pay: No charge	1.145 [0.430–3.047]	1.325 [0.593–2.964]	0.780 [0.445–1.365]
	Pay: Other	1.047 [0.777–1.411]	0.750 [0.531–1.059]	0.830 [0.708–0.974]
ZIPINC (Ref: 1st–25th perc.)	ZIPINC: 26–50th perc.	0.981 [0.869–1.106]	0.807 [0.720–0.905]	1.050 [0.984–1.120]
	ZIPINC: 51st–75th perc.	0.801 [0.708–0.906]	0.659 [0.585–0.742]	1.064 [0.998–1.134]
	ZIPINC: 76–100th perc.	0.838 [0.741–0.949]	0.6363 [0.563–0.718]	1.048 [0.982–1.119]
MetS (Ref: 0)		1.739 [1.575–1.920]	1.871 [1.703–2.055]	1.457 [1.381–1.537]
c-statistic		0.737	0.755	0.779
Goodman–Kruskal Gamma		0.486	0.521	0.561

**Table A3:** Association between metabolic syndrome (CMS > 2) and cardiovascular outcomes in patients with Crohn’s disease.

		Odds Ratio Point Estimate [95% CI]		
Covariates		ACS	HF	Arrhythmia
Age		1.050 [1.047–1.053]	1.045 [1.042–1.048]	1.060 [1.059–1.062]
Race (Ref: White)	African American	0.780 [0.659–0.922]	1.224 [1.082–1.385]	0.821 [0.755–0.893]
	Hispanics	0.852 [0.682–1.065]	0.843 [0.692–1.027]	0.656 [0.579–0.742]
	Asian or Pacific Islander	1.059 [0.660–1.700]	0.615 [0.367–1.032]	0.715 [0.557–0.918]
	Native American	1.547 [0.836–2.864]	1.543 [0.956–2.492]	0.843 [0.546–1.302]
	Other Ethnicity	1.059 [0.780–1.437]	0.990 [0.746–1.313]	0.915 [0.779–1.075]
Female (Ref: Male)		0.717 [0.661–0.777]	0.964 [0.896–1.038]	0.754 [0.724–0.786]
Hospital Census Division		0.987 [0.970–1.006]	1.041 [1.025–1.056]	1.001 [0.992–1.010]
Primary Expected Payer (Ref: Medicare)	Pay: Medicaid	1.218 [1.024–1.448]	1.160 [0.997–1.350]	0.891 [0.810–0.980]
	Pay: Private insurance	1.029 [0.909–1.165]	0.536 [0.471–0.610]	0.843 [0.793–0.895]
	Pay: Self-pay	1.557 [1.194–2.030]	0.649 [0.475–0.886]	0.761 [0.639–0.906]
	Pay: No charge	0.716 [0.228–2.249]	1.155 [0.592–2.253]	0.657 [0.382–1.131]
	Pay: Other	1.332 [1.030–1.722]	0.963 [0.759–1.222]	0.860 [0.739–1.001]
ZIPINC (Ref: 1st–25th perc.)	ZIPINC: 26–50th perc.	0.857 [0.766–0.960]	0.842 [0.766–0.925]	1.019 [0.960–1.081]
	ZIPINC: 51st–75th perc.	0.840 [0.750–0.941]	0.691 [0.625–0.764]	1.054 [0.993–1.118]
	ZIPINC: 76–100th perc.	0.686 [0.606–0.776]	0.576 [0.515–0.645]	1.058 [0.995–1.126]
MetS (Ref: 0)		1.726 [1.556–1.914]	2.099 [1.926–2.287]	1.586 [1.505–1.671]
c-statistic		0.747	0.765	0.788
Goodman-Kruskal Gamma		0.510	0.543	0.580

**Table A4:** List of abbreviations used in the manuscript.

ACS	Acute coronary syndrome
AHRQ	Agency for Healthcare Research and Quality
AZA	Azathioprine
BMI	Body mass index
CD	Crohn’s disease
CMS	Calculated metabolic score
CV	Cardiovascular
CVD	Cardiovascular disease
DLD	Dyslipidemia
DM	Diabetes mellitus
GI	Gastrointestinal
HCUP	Healthcare Cost and Utilization Project
HDL	High density lipoprotein
HF	Heart failure
HTN	Hypertension
IBD	Inflammatory bowel disease
ICD 10	International Classification of Disease, 10th Edition
IDF	International Diabetes Federation
IRB	Institutional Review Board
MAFLD	Metabolic-dysfunction-associated fatty liver disease
MASLD	Metabolic-dysfunction–associated steatotic liver disease
MetS	Metabolic syndrome
MI	Myocardial infarction
NIS	National Inpatient Sample
OR	Odds ratio
UC	Ulcerative colitis
WHO	World Health Organization
